# Observations on Lacerated and Contused Wounds

**Published:** 1914-08

**Authors:** L. Sexton

**Affiliations:** Tulane


### TEXAS MEDICAL JOURNAL

Dr. F. E. Daniel, Founder. Established July, 1885

MRS. F. E. DANIEL, - Publisher and Managing Editor

Published Monthly.—Subscription, $1.00 a Year.

VOL. XXX AUSTIN, AUGUST, 1914 No. 2

The publisher is not responsible for views of contributors

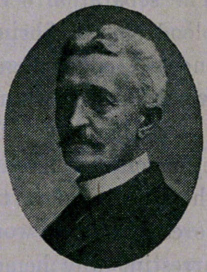

### Original Articles.

For Texas Medical Journal.

### Observations on Lacerated and Contused Wounds.

BY L. SEXTON, B. S., M. D., OF TULANE.

The most common wounds calling for treatment by the surgeon and general practitioner in the country and smaller towns, as well as the city, are of the above variety, and such a radical change in their treatment and management has taken place, that it may be of interest to note the present, in contradistinction to the past methods of caring for such wounds.

These injuries, are solutions in the continuity of the soft parts, produced by a dragging force or trauma with blunt instruments, blows and machinery. Infection with bacteria usually takes place at the same time the wound is produced, through dust, oil, cinders, dirt and machinery grease, which is often ground into the tissue at the time. These wounds bleed less than the incised, because the vessels are torn or twisted, the torn and irregular edges favors the coagulation of blood. The gaping or separation from such injuries are not so marked as in incised wounds, the tissue is often crushed and pulpified. The pain from such wounds is dull, throbbing and aching if the nerve is not divided, but if pressed upon near the crushed parts, the pain is continuous until the nerve is divided or released. From the above fact contused wounds are more painful than incised wounds, but bleed less.

Shock is largely dependent upon the place of the blow and the sensibility of the patient, and the amount of crushing injury; in avulsion and crushing of bone, of both limbs, as in railroad accidents, the shock may prove fatal. Healing in very slight contused wounds of this kind may take place without much inflammation. The separation between the tissue is filled with small coagulum or blood clot of fibrin, which acts as nature's sticking plaster, bringing the edges together. This fibrin forms a thin scab over the wound under which healing takes place.

The majority of contused and lacerated wounds, however, heal by second rather than first intention. The first desideratum in all these injuries is to get rid of whatever infection that has been forced into the wound at the time of the injury, at the same time arresting any hemorrhage which may be present and approximating the parts as snugly as possible. It should always be remembered that reactionary hemorrhage may take place in lacerated or contused wounds when the temporary plug that stopped the vessel is blown out by reaction, or we may have secondary hemorrhage, which may result from sloughing tissue, including the veins and arteries; hence, it is important that all risks of hemorrhage from these sources be attended to at the first dressing. $H_2O_2$ (Hydrogen Peroxide) diluted half with sterile hot water, poured freely into such wounds with the flaps held up so that all the crevices of the wound may be filled, aids in boiling out the foreign particles of dirt and infection and at the same time it acts as a good hemostatic. The use of the Peroxide of Hydrogen at subsequent dressing may be questioned, particularly if delicate epithelium has begun to cover the wound. All applications should be very mild at this stage of healing. After washing out the wound with sterile water, all pulpified tissue and skind that is known to be dead from lack of blood supply and comminution had as well be removed with forceps and scissors, as to be left to slough and infect the wound later.

It is proper to state here that owing to the abundant blood supply to the hands and feet that many apparently destroyed extremities have been saved by conservative surgery. The recuperative power of nature in mending these members should always be given a chance. You can amputate later if the member is destroyed, but you can never retrieve the mistake of amputating too early.

One of the means of arresting hemorrhage from these wounds is by pouring an abundant supply of hot water into the wound, which acts by flushing out the foreign bodies driven into the wound, also constricting the blood vessels and arresting hemorrhage. If these crushed wounds are over bony prominences, compression is better for arresting hemorrhage than putting in unnecessary ligatures which increase the risk of infection. Sterile gauze pressed into the wound as compress or held with firm pressure under digital compression checks the average bleeding and pain within a short space of time. Where a larger artery is concerned a ligature or suture is required. Fingering in the wound or further traumatising the tissue should not be allowed. Hemostatic forceps and tortion will help to control the smaller vessels better than ligatures, which might carry infection. Chemical styptics have no place in the arrest of hemorrhage in these wounds, as they destroy all chance of union by first intention by the introduction of a foreign body into the wound, so they are only mentioned to be condemned. Their use may be permitted upon malignant or sloughing wounds.

The treatment of all non-operative wounds resolves itself into cleansing, not only the wound, but fixing the cells and epithelium, and cleansing as well the tissue adjacent to the wound, not by washing and scrubbing with strong soaps, as formerly done, but by wiping away from the wound (rather than washing the wound) with such substances as chemical solvents to the grime or grease. The grease or oil from a machine accident can best be removed by first applying the half alcohol and Tr. iodine directly into and around the wound, then cleansing the wound by wiping away from its edges with sterile gauze saturated with turpentine, benzine or gasoline.

The infection by this method is much rarer than when the injured part was scrubbed with soap and water, in a vain effort to cleanse a hand, into which an accumulation of grit and stain had been ground in for months or years. Wet carbolic or bichloride dressing for first twenty-four hours to remove grime is an accepted procedure.

The preliminary painting of the parts with alcohol and Tr. iodine seems to destroy all the skin germs. If it is a hairy portion of the body that is injured, it may be soaped and shaved away from the wound. Not only are all foreign bodies to be removed at the first dressing, but also particles of crushed bone if devoid of periosteum is likely to act as foreign body, and should be removed, as should all other thoroughly dead tissue.

The future function of fingers have been greatly benefited by suturing divided tendons, muscles and nerves to their mates. It is best to slit up pockets of wounds, if necessary to gain entrance into crannies where foreign bodies may have lodged; mopping out the thoroughly exposed floor of the wound with equal parts of Tr. iodine and alcohol. Placing gauze drains into the deeper recesses, is a safe method against infections. The sutures in such wounds should be interrupted and tied loosely, so as to hold the edges of the parts together and to admit of free drainage, as this is imperative particularly in large lacerated wounds. If the soft parts are not so pulpified as to make dead and living tissue look alike, a moist antiseptic drip from an irrigator over the injured part, which is supported by a Kelly pad, is a good procedure for twenty-four or forty-eight hours until it can be determined what can be saved, and what is irretrievably lost. This drip should be kept warm and mildly antiseptic, a saline solution or large moist warm dressings may be substituted for this drip over the injured part.

The redressing, if the bandages are not much soiled, no pus or pain present, need not be repeated until the second or third day. The rule of dressing such wounds daily is a mistake unless they are infected, sloughing or very painful. Fever is the forerunner of general infection or cellulitis, which occasionally follow such wounds.

When wounds are already seriously infected before they come to the doctor, which is often the case, the treatment by saline drip, 1-5000 bichloride or one-half of 1 per cent carbolic, until all suppuration has stopped and inflammation subsided, is to be followed by dusting powder of subiodide of bismuth, or one-half and one-half alcohol and tincture iodine, then covered by sterile gauze.

The treatment of such wounds by pure carbolic acid, followed immediately by alcohol to neutralize the acid, has not been followed much, as it is considered too heroic.

Coaptation of the wounds is essential for their ready healing; a wound well sewed is half healed. All sutures should be put in loose to prevent tissue necrosis after the wound swells. Many times a gaping wound can be pretty well approximated by the Z. O. plaster. When it becomes necessary to use sutures they should be interrupted and of silk worm, if the wound is to be kept moist all the time to prevent absorption before the wound is healed. We have used retention sutures to good effect where they held granulating edges together.

Draining from the most dependent part of the wound is very important. Stabbed wounds through the skin on opposite side of limb often facilitates this drainage. In these large wounds, where the question of infection is not settled, primary drainage and secondary sutures, i. e., sutures not tied when first put in but left loose to be tied when risk of infection has passed and the drainage gauze removed. Gauze drains or rubber or glass perforated tubes may be used for the first forty-eight hours to be removed at the second dressing of the wound.

Rest is next in importance to asepsis in these cases of injury, and to obtain it it becomes necessary to keep the patient in bed, arm in sling, or limb or arm in splints. Immovable dressings that prevent muscular action reduce the amount of pain and promote healing of the wound. All limbs with large lacerated wounds should be immobilized by bandages, splints or other methods.

The routine injection with 1500 units anti-tetanic serum in all these lacerated wounds has become the rule in busy surgical clinics. Whether less tetanus is due to the injections of the serum, or to better cleanliness and dressing methods, it is impossible to say. The serum has no curative property after tetanus has developed, it is only supposed to be preventive.

The above methods have been applied in the largest clinic in the Charity Hospital in New Orleans for the past few years with the best results and fewest infections.

